# Knowledge and practice of the physical activity prescription by generalists and specialist physicians of the city of Kinshasa: a cross-sectional study

**DOI:** 10.1186/s13102-023-00737-w

**Published:** 2023-09-28

**Authors:** Emeka Bowamou Christian-Khalifa, Nkodila Natuhoyila Aliocha, Nkiama Ekisawa Constant, Divengi Nzambi Jean-Paul, Kintoki Vita Eleuthère, Longo-Mbenza Benjamin, M’buyamba-Kabangu Jean-Réné, Kianu Phanzu Bernard

**Affiliations:** 1 Faculty of Medicine, Department of Physical Medicine and Rehabilitation, University of Kinshasa, Kinshasa, Democratic Republic of the Congo; 2 School of Public Health, Department of Statistics, University of Kinshasa, Kinshasa, Democratic Republic of the Congo; 3 Department of Internal Medicine, University of Kinshasa, Kinshasa, Democratic Republic of Congo; 4 Cardiology Unit, University Hospital of Kinshasa, University of Kinshasa, Kinshasa, Democratic Republic of Congo

**Keywords:** Contributing factors, Health professionals, Obstacles, Physical activity

## Abstract

**Background:**

The health benefits of regular physical activity (PA) are well documented. However, several people in both developed and developing countries do not meet PA recommendations. Health professionals are believed to be potential PA promoters. The purpose of this study is to gain insight into general and specialist practitioners’ knowledge, practices and PA prescription-related factors in private and public hospitals in Kinshasa.

**Methods:**

A multicenter cross-sectional analytical study was conducted among general and specialist practitioners in the Democratic Republic of the Congo’s capital using a declarative and anonymous questionnaire.

**Results:**

Overall, 40.2% of the participants were interested in their patient’s PA, 2.3% prescribed PA, and 0.9% did it correctly. Specialist physicians (SPs) prescribed PA more frequently than general practitioners (GP), and private hospital physicians prescribed PA more frequently than public hospital physicians. Five factors were independently associated with participants in prescribing PA: being in a private hospital increased the likelihood of prescribing PA by twofold (aOR, 1.83; 95% CI, 0.99–3.39; *p* = 0.055), being an SP increased the likelihood by sixfold (aOR, 6.22; 95% CI, 3.78–10.51; *p* = 0.000), being an internist increase the likelihood by sixfold (aOR, 5.81; 95% CI, 3.45–9.78; *p* = 0.000), being cardiologist by a factor of 12 (aOR, 12.91; 95% CI, 4.37–38.15; *p* = 0.000) and knowing the benefits of PA by a factor of 2 (aOR, 2.29; 95% CI, 1.29–4.08; *p* = 0.006). The most common reason given for a lack of interest in patients’ PA, followed by a lack of knowledge about current PA prescribing recommendations and a lack of time.

**Conclusions:**

SPs and professionals in the private health sector were the most interested in their patients’ PA. A small portion of them actually prescribed it, and only a tiny proportion did it correctly. This bleak picture highlights a need to rethink the undergraduate medical curricula, especially about teachings on the importance and use of PA as a medicine in its own right in disease prevention and treatment.

## Background

Regular physical activity (PA) has been shown to be beneficial in the areas of all-cause mortality, cancer, cardiovascular health, musculoskeletal health, metabolic health and neurocognitive health [1]. As a result, PA is now regarded as a standalone therapeutic in the primary, secondary, and tertiary prevention of chronic pathologies. Despite this, recent global estimates show that one in every four adults [2] and four out of every five adolescents [3] are insufficiently physically active, emphasizing the importance of population-wide initiatives to increase their level of PA.

Brief interventions in primary care settings, according to randomized controlled trials, effectively improve patients’ PA [4, 5]. Beyond brief interventions, primary care professionals have been shown to be one of the most cost-effective ways to increase PA prescription [6]. As a result, health professionals have been identified as potential promoters of PA [7, 8]. Some scientific societies advocate incorporating PA promotion into the routine clinical practice of primary care physicians (GP) [9].

So far, studies evaluating the participation of health professionals in promoting PA have primarily focused on GPs. The few studies involving specialists physicians (SPs) have not compared these specialists and GPs. Ezgi Agadayı et al. (10) compared SPs and research assistants of family medicine but not with GPs. They found a statistically significant difference in PA knowledge and prescription between these groups. Furthermore, to our knowledge, studies comparing physicians from private hospitals (PrH) with those from public hospitals (PH) are almost nonexistent. This information would be important to consider measures for improving general and specialist practitioners from different health sectors participating in PA promotion. Therefore, the hypothesis of this research was that a physician’s sector of activity, qualification, field of specialization, and knowledge of physical activity promotion recommendations were all factors that could influence their knowledge and practice of prescribing physical activity, among other parameters. As a result, the first goal of this study was to describe and compare the knowledge and practices of GP and SP in the PrH and PH, and the second goal was to identify the factors that lead physicians to prescribe PA to their patients.

## Methods

### Study design

This cross-sectional analytical study was conducted between April 1 and June 30, 2022, in both PH and PrH of the city-province of Kinshasa. From the Ministry of Health registers, 5 PrH and 5 PH were randomly selected. The participants were randomly selected from the lists of physicians from the selected hospitals.

### Sample size calculation

A sample size of 367 was estimated taking the population size of 8000 physicians registered with the National Medical Council, working in the Province City of Kinshasa (confidence level = 95% and margin of error = 5%).

### Participant selection

Participants in this study had to be a GP or SP registered with the National Medical Council, working in the Province City of Kinshasa and willing to answer the study questionnaire. Participants who did not complete or returned the questionnaire were excluded from the study.

**Fig. 1:**
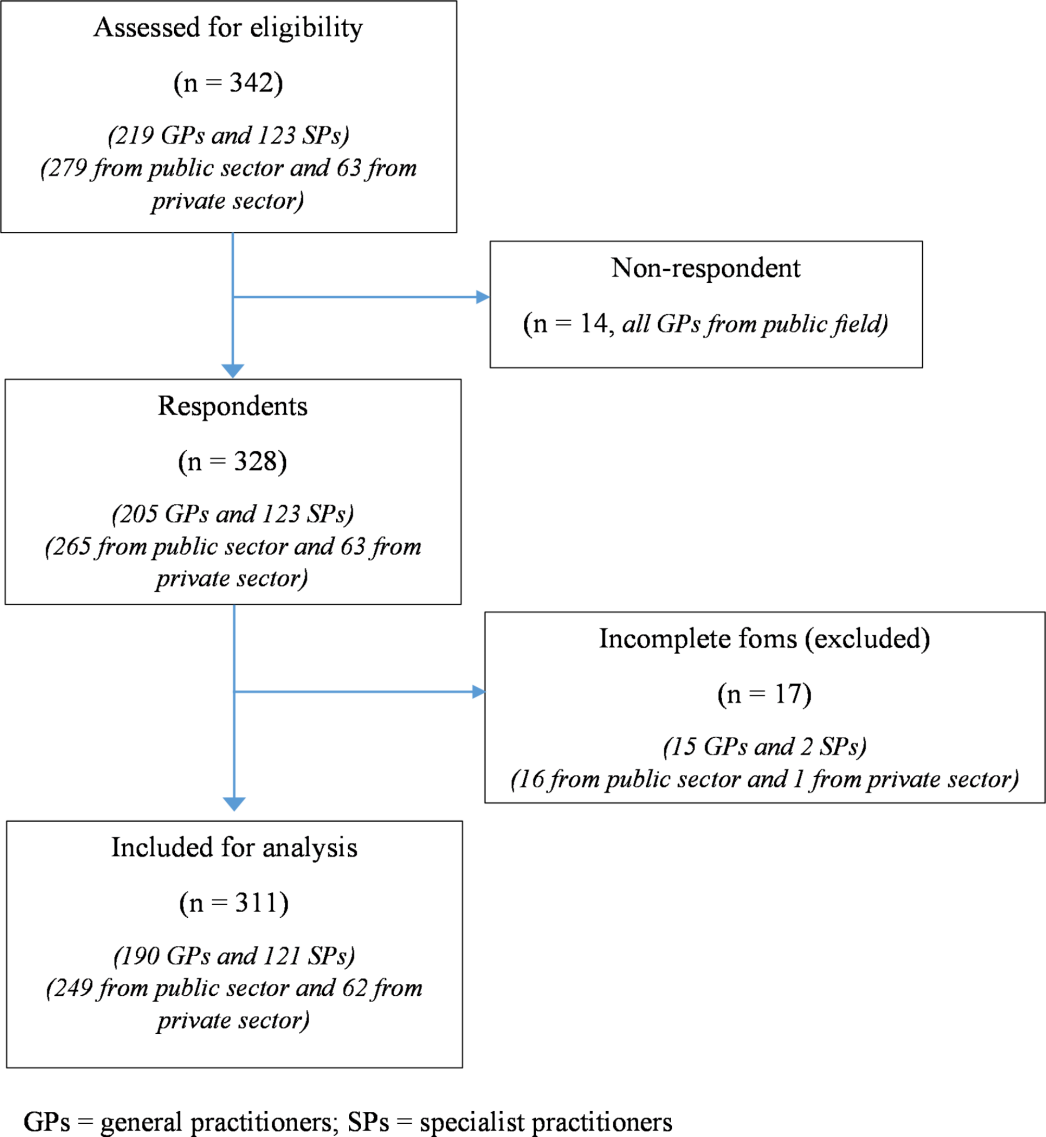
Study flowchart

In the beginning, 342 people were chosen to participate in the study, including 279 from PH and 63 from PrH. There were 123 SPs and 219 GPs among them; 14 were lost during follow-up (did not submit the completed questionnaire) and 17 were excluded due to incomplete questionnaires. The selection process is summarized in Fig. 1.

### Study procedures

A declarative and anonymous questionnaire was created in collaboration with physical medicine specialists and the statistics department of Kinshasa’s School of Public Health. This questionnaire included 21 open or closed questions with single or multiple-choice answers based on international PA prescription recommendations. It has been designed to have a response time of no more than 5–10 min. The respondent’s age and gender, qualifications, area of practice (PrH or PH), knowledge of PA practice and prescribing recommendations and barriers to prescribing PA were all questioned. The questionnaire had previously been tested with eight participants: two PrH GPs, two PH GPs, two PrH SPs, and two PH SPs. This pre-test made it possible to improve and refine the questionnaire, considering some difficulties encountered by the participants and statistician for collected data exploitation.

Selection biases were minimized through the following precautions: the sampling was probabilistic, and the nonresponse rate was globally minimized by the interest aroused by the study and the sufficiently long delay (24 h with possible extension up to 48 h) given to everyone to complete their questionnaire.

To minimize classification bias, the protocol was developed considering various precautions: the concepts were clearly defined, and highly confidential data were avoided. To minimize nonresponse, a prior appointment was obtained, the participants had firm promise of confidentiality, and the participants were free to choose the moment and place (hospital or home) to complete their questionnaire under the least stressful conditions possible. In addition, essential cooperation of each participant was reiterated for the success of the survey.

Following the usual administrative approvals, a team of trained researchers comprised finalists was mobilized to contact the physicians of the selected hospitals. The questionnaire was a two-page hard copy that was personally delivered to each physician who met the eligibility criteria. The same researcher was in charge of retrieving and delivering the completed questionnaire from the respondent to the principal investigator. When a participant did not submit their completed questionnaire within 24 h, they were given another 24 h. If the questionnaire was not returned within 24 h, the candidate was dropped from the study.

### Operational definitions

In this analysis, the following definitions were used:

- PA interest was defined by responding ‘always’ or ‘often’ to the question, ‘do you ask your patients if they participate in regular PA?’
- Non-interest in PA was defined as ‘rarely’ or ‘never’ responding ‘rarely’ or ‘never’ to the question ‘Do you ask your patients if they engage in regular PA?’
- Knowledge of PA benefits was defined by marking all proposed PA benefits claims.
- When participants flagged all statements about PA indications, they were considered to be aware of them.
- A correct PA prescription was defined as one that includes information on the frequency, duration, intensity, and type of activities to be performed.
- Good knowledge of weekly PA volume recommendations was defined by verifying the first statement related to this question (150 min of moderate-intensity PA per week).

### Statistical analysis

EpiInfo7 software was used to enter data into a computer, and SPSS Statistics for Windows (IBM SPSS Statistics for Windows, Version 27.0. IBM, Armonk, NY) was used to perform all statistical analyses. Tables or graphs were used to present results as needed. Continuous quantitative variables with Gaussian distributions were presented as mean ± *SD*, whereas those with non-normal distributions were presented as median (extreme). The qualitative variables were expressed as a percentage. The Chi-square test, Student *t* test, and Mann–Whitney Wilcoxon test were used to compare proportions, medians, and means, respectively. Logistic regression using the step-by-step method (backward method) was used to identify independent determinants of PA prescription. The variables that emerged significantly in the bivariate analysis were retained in the multivariate model. Collinear variables (specialists, internists, and cardiologists) were separately introduced into the multivariate analysis. Thus, three models were made based on collinear variables. In the first model (health sector category), internist and knowledge about the benefits of PA were the covariates. In the second model (health sector category), cardiologist and knowledge about the benefits of PA were the covariates. Finally, in the third model (health sector category), the specialist and knowledge about the benefits of PA were the variables. Odds ratio (OR) and adjusted odds ratio (aOR) with their confidence intervals were calculated to estimate the degree of association. A *p* value of < 0.05 was considered statistically significant.

### Ethical considerations

The questionnaire was administered anonymously, in accordance with the Helsinki III Declaration, and the information obtained was used while respecting the respondents’ confidentiality and privacy. The participants’ data were also manipulated and statistically anonymously and confidentially. Study approval was obtained with waiver of written informed consent from the « comité national d’éthique de la santé » (National Health Ethics Committee), No. 415/CNES/BN/PMMF/2022.

## Results

### General characteristics of the study population

The study population consisted of 311 physicians, including 215 men and 96 women, with a sex ratio of 2.2 (in favor of men).

### Sociodemographic characteristics of the study population

The sociodemographic characteristics of the study population as a whole and by sector of practice are shown in Table 1. No statistically significant differences were found in sex or age range based on the health sector (PH or PrH). 190 (61.1%) participants were GPs. SPs were statistically more represented in PrH than in PH [39 (62.9%) vs. 82 (32.9%), p value < 0.001], whereas GPs predominated in PH than in PrH [167 (67.1%) vs. 23 (37.1%), p value < 0.001].

**Table 1:** Sociodemographic characteristics of the study population by sector of practice

Variables	Sector of practice			
	*N* = 311	Public (*n* = 249)	Private (*n* = 62)	*p* value
Sex				0.334
Male	215 (69.1)	174 (69.9)	41 (66.1)	
Female	96 (30.9)	75 (30.1)	21 (33.9)	
Age, years	43.8 ± 11.2	41.6 ± 10.3	44.2 ± 11.8	0.269
Age group				
25–29	42 (13.5)	30 (12.0)	12 (19.4)	0.125
30–39	93 (29.9)	74 (29.7)	19 (30.6)	0.987
40–49	101 (32.5)	79 (31.7)	22 (35.5)	0.589
50–59	56 (18.0)	49 (19.7)	7 (11.3)	0.098
≥ 60	19 (6.1)	17 (6.8)	2 (3.2)	0.158
Qualification				**< 0.001**
GPs	190 (61.1)	167 (67.1)	23 (37.1)	
SPs	121 (38.9)	82 (32.9)	39 (62.9)	
Area of specialization				**0.001**
Internal medicine	46 (14.8)	26 (10.4)	20 (8.0)	**< 0.001**
Cardiologist	13 (4.2)	6 (2.4)	7 (2.8)	0.489
Other IM specialists	33 (10.6)	**20 (**8.0**)**	**13 (21.0)**	**0.011**
Gynecology obstetrics	23 (7.4)	17 (6.8)	6 (9.6)	0.122
Surgery	20 (6.4)	14 (5.6)	6 (9.6)	0.291
Pediatrics	25 (8.0)	18 (7.2)	7 (11.2)	0.117
Others	7 (2.2)	7 (2.8)	0 (0.0)	
Internist				0.158
Cardiologists	13 (4.2)	6 (2.4)	7 (18.0)	
Other internists	33 (10.6)	20 (8.0)	13 (21.0)	

IM, internal medicine; GPs, general practitioners; SPs, specialist practitioners

### Sociodemographic characteristics of the study population by participant qualification

As shown in Table 2, no statistically significant difference was found between GPs and SPs with respect to sex (p value = 0.111) and age (p value = 0.247). GPs and SPs were distributed in similar proportions (p value = 0.051) in the PH and PrH.

**Table 2:** Sociodemographic characteristics of the study population by participant qualification

Variables		Participant qualification		
	*Overall* *n = 311*	Generalists (*n* = 190)	Specialists (*n* = 121)	*p* value
Hospital				**0.051**
Public	249 (80.1)	167 (87.9)	82 (67.8)	
Private	62 (19.9)	23 (21.1)	39 (32.2)	
Sex				0.111
Male	215 (69.1)	126 (66.3)	89 (73.6)	
Female	96 (30.9)	64 (33.7)	32 (26.4)	
Age, years	43.8 ± 11.2	43.8 ± 11.2	40.6 ± 11.3	0.247
Age groups, years	43.8 ± 11.2	40.6 ± 11.3	43.9 ± 11.6	0.269
25–29	42 (13.5)	28 (14.7)	14 (11.6)	0.208
30–39	93 (29.9)	55 (28.9)	38 (31.4)	0.172
40–49	101 (32.5)	57 (30.0)	44 (36.4)	0.199
50–59	56 (18.0)	34 (17.9)	22 (18.2)	0.559
≥ 60	19 (6.1)	16 (8.4)	3 (2.5)	0.090

### Participants’ interest in patients’ PA according to qualification

Table 3 illustrates the proportions of participants who are interested in the PA of their patients and those who evaluate the level of this activity and the tools used for this evaluation, according to the qualification of the participant (GPs vs. SPs). As shown in this table, only 125 (40.2%) participants were interested in the PA of their patients. Compared with GPs, SPs were more interested in it (< 0.001). In addition, only 36 (11.5%) participants used specific tools to assess the PA level of their patients, and this proportion was statistically comparable among GPs and SP (p value = 0.182). The pedometer was the tool most often used by GPs, followed by the self-report activity diaries, whereas SPs most often used the self-report activity diaries. However, except for the pedometer, which was used significantly more by GPs than by SPs (p value = 0.025), other PA assessment tools were used in statistically similar proportions by both.

**Table 3:** Participants’ interest in patients’ PA according to qualification

Variables		Participants’ qualification		
	*Overall* *n = 311*	Generalists (n = 190)	Specialists (n = 121)	*p* value
Participants interested in patients’ PA	125 (40.2)	29 (15.3)	96 (79.3)	**< 0.001**
Using tools	36 (11.5)	19 (10.0)	17(14.0)	0.182
Frequently used tools				
Marshall questionnaire	5 (1.6)	1 (0.5)	4 (3.3)	0.156
IPAQ	3 (1.0)	2 (1.05)	1 (0.8)	0.223
Self-report activity diaries	15 (4.8)	6 (3.15)	9 (7.4)	0.094
Pedometer	14 (4.5)	10 (5.2)	4 (3.3)	**0.025**

### Participants’ interest in patients’ PA according to Health sector

Table 4 illustrates the proportions of participants who were interested in the PA of their patients and of those who evaluate the PA level of their patients and the tools used for this evaluation, according to the health sector (pH vs. PrH). As shown, PrH participants were more interested in the PA of their patients and more often used specific tools to assess their PA levels than PH participants.

In addition, the tools used varied significantly depending on the health sector; the pedometer and the IPAQ Questionnaire were being used more in PH than in PrH, whereas the Marshall questionnaire and self-report activity diaries were more often used in PrHr than in PH.

**Table 4:** Participants’ interest in patients’ PA according to health sector

Variables		Health sector		
	**Overall** **(311)**	**Public** **(n = 249)**	**Private** **(n = 62)**	**p value**
Participants’ interest	125 (40.2)	75 (30.1)	50 (80.6)	< 0.001
Using tools	36 (11.6)	24 (9.6)	12 (19.4)	**0.032**
Frequently used tools				
Marshall questionnaire	5 (1.6)	1(0.4)	4 (6.4)	**0.026**
IPAQ	3 (1.0)	1 (0.4)	2 (3.2)	**0.043**
Self-report activity diaries	15 (4.8)	11 (4.4)	4 (6.4)	**0.030**
Pedometer	14 (4.5)	12 (4.8)	2(3.2)	**0.003**

IPAQ, International Physical Activity Questionnaire

### General knowledge and prescription of PA according to participants’ qualification

Table 5 summarizes the main information concerning the knowledge and practices of the participants in terms of promoting PA according to their qualification (GPs vs. SPs).

Only 77 (24.8%) participants had a good knowledge of the benefits of PA. No statistically significant difference was found between GPs and SPs in this knowledge of the benefits of PA (p value = 0.068).

Table 5 also indicates that weight loss, followed by a reduction in cardiovascular risk and improvement in the quality of life, are the benefits of PA that were most recognized by the study participants. Apart from weight loss, which was recognized as a potential benefit of PA in a similar proportion of GPs and SPs, other potential benefits of PA were more often recognized by SPs than by GPs.

Concerning the knowledge of the average weekly duration recommended for the practice of PA, only 137 (44%) participants were informed about it, and considerably more SPs than GPs were informed about it.

In general, obesity, followed by hypertension and diabetes mellitus were situations that were most often identified by the participants as requiring PA prescription. These same situations, in addition to being elderly and living with disabilities, were more often identified by SPs than by GPs.

As shown in Tables 5 and 120 (38.6%) participants, which were more often SPs than GPs, gave instructions in relation to the practice of PA. These instructions were more often given orally, and only 7 (2.2%) participants provided them as a medical prescription. The frequency of formulation of these instructions in oral or written form (medical prescription) and the frequency of correctly formulated prescriptions were comparable between generalists and specialists.

Forgetting and lack of knowledge about the recommendations regarding PA prescription were the reasons most often cited to justify PA nonprescription by all participants. Forgetting was more often mentioned by SPs than by GPs, whereas more GPs often mentioned a lack of knowledge about recommendations.

**Table 5:** General knowledge and prescription of PA according to participants’ qualification

Variables	All n = 311	Generalist n = 190	Specialist n = 121	p value
Knowledge of PA benefits	77 (24.8)	41 (21.6)	36 (29.8)	0.068
Type of known benefits				
Improve the quality of life	188 (60.5)	104 (54.7)	84 (69.4)	**0.007**
Reduce cardiovascular risk	251 (80.7)	156 (76.8)	105 (86.8)	**0.020**
Lose weight	282 (90.7)	173 (91.4)	109 (90.1)	0.461
Maintain weight loss	90 (28.9)	48 (25.3)	42 (34.7)	**0.049**
Type of patients identified by participants as requiring a PA prescription				
Obese	263 (84.6)	145 (76.3)	118 (97.5)	**< 0.001**
Hypertensive	182 (58.5)	85 (44.7)	97 (80.2)	**< 0.001**
Diabetic	143 (46.0)	61 (32.1)	82 (67.8)	**< 0.001**
Pregnant women	41 (13.2)	22 (11.6)	19 (15.7)	0.190
Menopausal women	16 (5.1)	7 (3.7)	9 (7.4)	0.116
Old man	74 (23.8)	38 (20.0)	36 (29.8)	**0.034**
Persons with disabilities	10 (3.2)	1 (0.5)	9 (7.4)	**0.001**
Practical instructions on PA	120 (38.6)	52 (27.3)	68 (56.2)	**0.001**
Types of instructions given to patients on PA				
Oral advice	120 (38.5)	52 (27.3)	68 (56.2)	0.001
Written advice (prescription)	7 (2.2)	2 (1.3)	5 (4.2)	0.186
Oral and written	7 (2.2)	2 (2.2)	5 (4.2)	0.186
Correct PA prescription	5 (1.6)	1(0.5)	4 (3.3)	0.109
Recommended weekly duration	137 (44.1)	18 (9.5)	32 (26.4)	**< 0.001**
Reasons for not prescribing PA				
Forgetting	165 (53.1)	90 (47.6)	75 (62.5)	**0.007**
Lack of awareness of current recommendations	130 (41.8)	99 (52.4)	31 (26.3)	**< 0.001**
Lack of time	86 (27.7)	48 (26.1)	38 (32.2)	0.154
Lack of motivation on your part	58 (18.6)	42 (22.2)	16 (13.3)	**0.034**
PA would throw my patient’s condition out of balance	20 (6.4)	13 (6.9)	7 (5.8)	0.456
I don’t believe in the benefits of PA	12 (3.9)	8 (4.2)	4 (3.4)	0.476

PA, physical activity

### General knowledge and PA prescription according to participants’ health sector

Table 6 summarizes the main information concerning the knowledge and practices of the participants in terms of promoting PA, according to their sector of medical activity (PH vs. PrH). No statistically significant difference in the awareness of PA benefits was found between PH and PrH participants. Moreover, a comparable proportion of PH and PrH participants recognized the various potential benefits of PA practice. A statistically greater proportion of PrH participants than PH participants was informed of the average weekly duration recommended for PA practice. Apart from obesity, which was more frequently identified by PrH participants than by PH participants as being a situation requiring PA prescription, all other situations were identified by a similar proportion of both participants. In addition, a statistically similar proportion of both participants gave practical instructions regarding PA practice. The frequency of the verbal or written form (medical prescription) of these instructions was also comparable among PH and PrH participants. However, PA prescription was more often correctly formulated by PrH participants than by PH participants. A lack of time was more often mentioned by PrH participants than by PH participants, whereas a lack of motivation was more often mentioned by PH participants. Other reasons that were cited to justify PA nonprescription were cited in similar proportions of PH and PrH participants.

**Table 6:** General knowledge and PA prescription according to participants’ health sector

Variables	Overall n = 311	Public sector n = 249	Private sector n = 62	p value
Knowledge of PA benefits	77 (24.8)	60(24.1)	17(27.4)	0.348
Type of known benefits				
Improve the quality of life	188 (60.5)	148 (59.4)	40 (64.5)	0.280
Reduce cardiovascular risk	231 (74.3)	198 (79.5)	53 (85.5)	0.189
Lose weight	282 (90.7)	227 (91.2)	55 (88.7)	0.350
Maintain weight loss	90 (28.9)	68 (27.3)	22 (35.5)	0.133
Recommended weekly duration	137 (44.1)	99 (39.8)	38 (61.3)	**0.002**
Patients identified by the participants as requiring PA prescription				
Obese	263 (84.6)	205 (82.3)	58(93.5)	**0.018**
Hypertensive	182 (58.5)	141 (56.6)	41(66.1)	0.112
Diabetic	143 (46.0)	112 (45.0)	31(50.0)	0.285
Pregnant women	41 (13.2)	33 (13.3)	8(12.9)	0.566
Menopausal women	16 (5.1)	14 (5.6)	2(3.2)	0.348
Older man	74 (23.8)	64 (25.7)	10(16.1)	0.075
Persons with disabilities	10 (3.2)	7 (2.8)	3(4.8)	0.319
Practical advice on PA	120 (38.6)	90 (36.1)	30 (48.3)	0.302
Types of PA instructions given to patients				
Oral advice	120 (38.6)	90 (36.1)	30 (48.3)	0.494
Written advice (prescription)	7 (2.3)	4 (1.6)	3 (4.8)	0.358
Oral and written	7 (2.3)	4 (1.6)	3 (4.8)	0.358
Correct PA prescription	5 (1.6)	3 (1.2)	2 (3.2)	0.911
Recommended weekly duration	137 (44.1)	99 (39.8)	38 (61.3)	0.002
Reasons of not prescribing PA				
Forgetting	165 (53.1)	132 (53.4)	33 (53.2)	0.544
Lack of awareness of current recommendations	130 (41.8)	110 (44.4)	20 (33.9)	0.094
Lack of time	86 (27.7)	61 (25.2)	25 (41.7)	0.010
Lack of motivation on your part	58 (18.6)	53 (21.4)	5 (8.2)	0.011
PA would throw my patient’s condition out of balance	20 (6.4)	17 (6.9)	3 (4.9)	0.417
I don’t believe in the benefits of PA	12 (3.9)	11(4.4)	1 (1.7)	0.284

PA, physical activity

### Determinants of PA prescription

Following univariate analysis (Table 7), five factors were significantly associated with participants prescribing PA: consulting in a PrH, being a specialist, being an internist, being a cardiologist and being aware of the PA benefits. After adjustments, three models were developed that demonstrated that being in a private hospital increased the likelihood of prescribing PA by twofold (aOR, 1.83; 95% CI, 0.99–3.39; p = 0.055), being an SP increased the likelihood by sixfold (aOR, 6.22; 95% CI, 3.78–10.51; p = 0.000), being an internist increase the likelihood by sixfold (aOR, 5.81; 95% CI, 3.45–9.78; p = 0.000), being cardiologist by a factor of 12 (aOR, 12.91; 95% CI, 4.37–38.15; p = 0.000) and knowing the benefits of PA by a factor of 2 (aOR, 2.29; 95% CI, 1.29–4.08; p = 0.006).

**Table 7:** Determinants of PA prescription

Variable	Bivariate analysis			Multivariate model 1			Multivariate model 2			Multivariate model 3	
	*p* value	OR (95% CI)		*p* value	aOR (95% CI)		*p* value	aOR (95% CI)		*p* value	aOR (95% CI)
Hospital sector											
Public		1			1			1			1
Private	0.004	2.29 (1.30–4.03)		0.203	1.51 (0.80–2.82)		0.055	1.83 (0.99–3.39)		0.252	1.45 (0.77–2.74)
Specialist											
No		1									1
Yes	< 0.001	6.72 (4.05–11.16)		—	—		—	—		< 0.001	6.22 (3.78–10.51)
Internist											
No		1			1						
Yes	< 0.001	6.27 (3.79–10.38)		< 0.001	5.81 (3.45–9.78)		—	—		—	—
Cardiologist											
No		1						1			
Yes	< 0.001	15.01 (5.14–43.77)		—	—		< 0.001	12.91 (4.37–38.15)		—	—
Knowing PA advantage											
No		1			1			1			1
Yes	0.001	2.34 (1.39–3.96)		0.005^*^	2.29 (1.29–4.08)		0.006^*^	2.19 (1.25–3.82)		0.006^*^	2.27 (1.27–4.05)

## Discussion

This study aims to describe physicians’ knowledge and practices regarding PA prescription in the Province City of Kinshasa.

This study is the first of its kind to highlight the influence of the health sector (private/public), qualification (generalist/specialist), and specialty area of physicians on their knowledge and practice of the physical activity prescription. Indeed, ours showed for the first time that SP and PrH physicians, on the other hand, were more interested in their patients’ PA than GP or PH physicians. On the other hand, being a specialist, internist, cardiologist and knowing the benefits of PA was significantly associated with prescribing PA.

In addition, the present study depicted that only a small percentage of participants (40.2%) were interested in their patients’ PA, and only 2.3% said they would recommend PA by medical prescription Furthermore, the present study showed that forgetting was the most frequently cited reason for not prescribing PA, followed by a lack of knowledge about current PA prescribing recommendations.

According to the literature, the percentage of physicians who are interested in their patients’ PA varies widely. One of the rare, if not the only, African studies have addressed the question of the prescription of PA focused on South African general practitioners (GPs). This study by Roos et al. (11) found substantially high prescription rates (90.9%). Possible reasons for this difference with our study may not only lie in possible self-report bias but also in the health systems differing between the Congolese and that of South-Africa. Outside Africa, a large study in Canada discovered that 85.2% of clinicians asked their patients about their PA habits (12). In Germany, 71.8% (13) to 90% (14) of GPs were said to be interested in the patients’ PA. According to Reimers et al. [15] more than 80% of neurologists polled in a nationwide study “frequently” counseled their patients on PA. Some authors addressed the issue of physicians’ interest in PA by examining the frequency with which patients reported receiving advice about PA from their doctors. According to a U.S. epidemiological study, 38% of this population received counseling that included a description of a specific activity [16]. Another study, also conducted in the United States, found that 34% of the patients surveyed had received PA advice from their family doctor during their most recent consultation [17].

The profile of the physicians and patients interviewed may explain the large disparity in the proportion of physicians interested in PA. In this study, respondents were physicians of various qualifications and specializations, both of PrH and PH, who cared for patients with various pathologies. Methodological diversity could also be considered: We used a hard-copy declarative and anonymous questionnaire delivered manually to each respondent, whereas others used a self-administered electronic questionnaire without the interviewers and respondents having ever physically met. Still, others proceeded by direct interview. It is also possible that the existence or absence of a public health policy governing doctors’ involvement of in PA promotion plays a significant role. It appears that the highest proportions are found in studies conducted in countries with public health policies, such as “prescription physical activity” (14, 18–24). Among other things, these guidelines give GPs the authority to prescribe PA. The lack of similar programs in the DRC could explain physicians’ low participation in promoting PA. Only 11% of the participants who were interested in their patients’ PA quantified it primarily using pedometers or self-report activity diaries. It is critical to assess patients’ PA levels because a dose-response relationship has been demonstrated with the risk of all-cause mortality and CVD morbidity and mortality in adults (25, 26). The pedometer’s popularity may be explained by its simplicity, low cost and ability to record short periods of PA (often missed by self-report measures) [27]. Furthermore, data from pedometers have been shown to correlate with biological outcomes [28, 29]. The pedometer counts steps and enables subjects to become aware of their activity from a simple PA, walking, which is accessible to the greatest number of people, in a utilitarian or leisure form [27, 30, 31]. For all these reasons, the pedometer is one of the most promoted means for PA objective evaluation [27], despite its drawbacks including not recording the intensity, frequency, or duration of PA [1, 2], inability to register PA involving horizontal movements that occur during periods of inactivity, leisure activities [3] and inducing reactivity in participants [4, 5].

Self-report diaries use real-time AP recording to collect the most detailed data [32], giving them an advantage over subjective declarative methods (questionnaires) [32, 33]. In general, objective assessment of PA using devices such as the pedometer are preferred over subjective methods, explaining the preference of GPs for the pedometer, which was the only device included in the assertions of the questionnaire used in this study. However, the self-report activity diaries, a subjective method that was used more by SPs than by GPs, was validated when it was compared with camera and accelerometer recordings [27, 34] or the pedometer [27, 35], despite its main limitation, which is the possibility of memory loss [36, 37].

Specialist physician appears to be more interested in the patient’s PA than GP in the current study. Furthermore, physicians in PrH, whether SP or GP, appear to be more concerned with their patients’ PA rather than PH, which has not been previously reported in the literature. This difference could be explained by doctors in PrH being more motivated (well paid) than those in the PH. When compared with GPs, SPs appeared to be more interested in PA and prescribed it more frequently and folded correctly than GPs. This discovery could be explained by SPs’ understanding of the benefits of PA, as well as increased remuneration. Aside from remuneration, lessons on nonpharmacological measures, particularly the role of PA in the treatment of chronic noncommunicable diseases, may not yet be well inserted in the curriculum of physician training in the Democratic Republic of the Congo.

In this study, 38% of the study participants gave practical instructions on PA, and only 2% formulated these instructions in the form of a prescription. This proportion is lower than that found in a Nigerian study conducted by Ale et al. [38], who discovered that three quarters of respondents prescribe PA. It is also lower than a survey of GPs in Catalonia, Spain, which found that 88% of doctors prescribe PA at least occasionally [39]. According to a 2001 Canadian survey, 69.8% of physicians prescribe PA in some way [12]. Compared with almost all the studies conducted elsewhere, the very low rate of prescription observed in this study would be due to the health system organization in the DRC, characterized by the absence of national guidelines on PA promotion compared with other countries, and insufficient sensitization among DRC doctors of the importance of PA prescription.

Only 0.9% appeared to get it right, specifying the type of exercise, intensity, duration, and frequency of the prescribed PA. To the best of our knowledge, this study is the first to look into these aspects of prescribing among the surveyed physicians. It was important to get an idea of how doctors prescribe PA, that is to say the content of their prescription, which should normally specify, as for a prescription of a pharmacological treatment, the type of PA to be practiced (molecule), its intensity (dosage), frequency (frequency of taking the drug), and duration of each session. The very low percentage of doctors who appeared to know the exact content of a PA prescription reflects a deficit in the training on PA prescription in the training course of the doctors surveyed.

On the contrary, five factors were found to be independently associated with the prescription of PA. Being a cardiologist was the most important determinant of prescribing PA, increasing the likelihood of prescribing PA by a factor of 12. Being an SP and an internist increased this likelihood by eightfold and knowing the benefits of PA increased it by sixfold, respectively. Other authors have mentioned the physician’s level of PA as a factor. Physicians who are physically active are more likely to recommend PA to their patients [40, 41].

The most common reason for not prescribing PA was forgetting, followed by a lack of knowledge about current PA prescribing recommendations and lack of time. Our findings on barriers to PA prescribing are consistent with those of other authors (41–47). Nauta et al. (47) identified forgetting as a major reason for not prescribing PA. Persson et al. (49) highlighted the current recommendations’ ignorance. Similarly, other previous studies have been identified a lack of time as a barrier to prescribing daily PA [41, 44, 45].

Consulting in PrH, being an SP, an internist, and a cardiologist, and knowing the PA benefits were the determinants of PA prescribing in this study. To our knowledge, no previous study has investigated the determinants of PA prescription. Consulting in PrH as a determinant of PA prescription probably reflects the effort often made in PrH to provide the best service to patients, mainly for commercial reasons. However, some studies have demonstrated better medical service in PrH than in PH [48, 49]. Being an SP as a determinant of PA prescription probably means that it is during specialist training that PA prescription is better taught than teaching in general medicine. Being a cardiologist as a determinant of PA prescription is probably due to the fact that the strongest evidence of the benefit of PA practice on health has been found in the field of cardiology [50].

### Limitations and strengths of the study

The interpretation of the findings of this study must take into account some methodological limitations, primarily the small sample size and the use of a declarative questionnaire. As a result, the current study’s findings may be skewed due to cognitive and/or affective bias. Despite its methodological limitations, this study has some strengths. This is the first study in RDC and Sub-Saharan Africa on physicians’ knowledge and practices in promoting PA. Almost all studies on the subject in the world have primarily focused mainly on GPs. By contrast, our research focused on the distinctions between GPs and specialists on the one hand and PrH physicians and PH physicians on the other.

## Conclusions

SPs and PrH professionals were the most interested in their patients’ PA levels. However, only a small percentage of them prescribed it, and an even tinier fraction did so correctly. This bleak picture highlights a need to (1) rethink the undergraduate medical curricula, especially about teachings on the importance and use of PA as a medicine in its own right in disease prevention and treatment in the daily clinical practice of Kinshasa’s physicians, [2] organize on-the-job training by the steering committees of both public and private hospitals in order to integrate the promotion of PA into the daily medical practice of physicians in the city of Kinshasa, [3] conduct future studies that directly observe physicians during medical consultations to avoid the inherent cognitive or affective biases of using declarative questionnaires,, [4] establish clear goals to improve the promotion of PA by physicians, and finally, [5] periodically assess progress.

## Data Availability

Data are available from the corresponding author (KPB) upon reasonable request.

## List of abbreviations

DRC: Democratic Republic of Congo
GPs: general practitioners
OR: odds ratio
PA: physical activity
PH: public hospital
PrH: private hospital
SPs: specialist practitioners
